# A multiobjective evolutionary algorithm for optimizing the small-world property

**DOI:** 10.1371/journal.pone.0313757

**Published:** 2024-12-03

**Authors:** Ruochen Zhang, Bin Zhu

**Affiliations:** 1 School of Economics and Management, Xi’an Shiyou University, Xi’an, Shaanxi, China; 2 School of Public Health and Emergency Management, Southern University of Science and Technology, Shenzhen, Guangdong, China; Sichuan University, CHINA

## Abstract

Small-world effect plays an important role in the field of network science, and optimizing the small-world property has been a focus, which has many applications in computational social science. In the present study, we model the problem of optimizing small-world property as a multiobjective optimization, where the average clustering coefficient and average path length are optimized separately and simultaneously. A novel method for optimizing small-world property is then proposed based on the multiobjective evolutionary algorithm with decomposition. Experimental results have proved that the presented method is capable of solving this problem efficiently, where a uniform distribution of solutions on the Pareto-optional front can be generated. The optimization results are further discussed to find specific paths for optimizing different objective functions. In general, adding edges within the same community is helpful for promoting ACC, while adding edges between different communities is beneficial for reducing APL. The optimization on networks with the feature of community structure is more remarkable, but community structure has less impact on the optimization when the internal community is triangles-saturated.

## 1. Introduction

Following the research of small-world and scale-free network models, many studies have been devoted to the analysis of structural properties of complex networks and social networks [1, 2]. In particular, promoting specific network structural indices has been a major focus, which is helpful in improving the efficiency of social system [3]. Among the different indices to be optimized, the small-world property is one of the most popular ones, and the optimization of which is of practical significance in various domains [4].

The term “small world” was first proposed by Milgram [5], who verified the small-world feature of human beings by conducting the famous experiment of six-degree separation. The small-world characteristics of social networks have been proved by a list of subsequent activities [6, 7]. Watts and Strogatz [1] then proposed a dynamic network model (WS model) to explain the small-world effect, which had a profound influence on complexity science and led the success of network science emerging as an interdisciplinary subject. In the rewiring process from the regular structure to the randomized state, they found there exists a critical phase where the network has higher average clustering coefficient (ACC) and lower average path length (APL), which are referred as the typical feature of small-world networks.

Small-world property emerges in various real-world domains, such as power grid [8], transport networks [9], control of epidemics [10], game theory [11] and management science [12, 13]. Improving the small-world property can promote the efficiency of signal transmission and accelerate the computational synchronization [4]. In the neural system, the interconnectedness of small-world network nodes can stimulate a list of biological properties [14–16]. When people fall into the prisoner’s dilemma, the small-world structure helps promote the information transmission of cooperative behaviors and speed up the cooperative convergence [11, 17, 18]. Since lower path length and higher clustering coefficient are helpful for spreading social information, the small-world network is beneficial to knowledge diffusion and knowledge transfer [19, 20]. The inherent strength of small-world topology has prompted research on how to efficiently generate small-world networks.

Since the process of rewiring edges may result in isolated nodes, Watts and Newman [21] proposed a novel model which is to adding shortcut edges. Similarly, Kleinberg [22] established the long-range connections based on the six-degree-separation theory. Subsequently, a list of studies have focused on introducing shortcuts to improve connectivity [23–28]. The optimal methods from these studies have been applied to various real-world situations, such as the set of wireless network [29]. However, most of the existing researches have paid much attention on the reduction of APL, which cannot fully represent the optimization of small-world property. A few studies have included other small-world characteristics, such as ACC [30, 31]. Even though, the main objective function is still the minimum of APL. Therefore, a research that cares about both APL and ACC is required.

Previous studies have found that the small-world structure facilitates the information diffusion and epidemiological transmission [32], and the mainstream reason for the conclusion is attributed to the small value of APL. In fact, reducing APL and increasing ACC have different effects on contributing to the improvement of network properties. Reducing APL lies in establishing relationships between two points over long distances, which highlights the strength of weak ties. Promoting ACC lies in strengthening the unity within the community, i.e., consolidating the effectiveness of strong ties. From a sociological perspective, both strong and weak ties have significant impacts on dynamic human behaviors, such as job seeking. Separately increasing ACC and decreasing APL are of great significance for optimizing different functions of social ties. Moreover, maintaining or adding social ties incurs costs, which requires seeking a balance between strong and weak ties within limited resources. The balance actually depends on the specific scenario in which the values of different objects are situated. For example, job seeking in family businesses relies on the function of strong ties, while it relies more on weak ties in multinational corporations. Simultaneously optimizing ACC and APL separately provides a new perspective for interpreting classic social theories like *The Strength of Weak Ties* [33], and it is also a new case for applying computational theory to social science.

In a recent study, Du et al. formulated the network property as ACC/APL [4], and proposed an optimization model to maximize the index. However, this model cannot control the ratios of the importance of clustering and distance. A scientific optimization for small-world property should separately consider the calculation of ACC and APL. The method of multiobjective optimization that seeks to optimize multiple objective functions helps us solve this problem. Schleich et al. proposed a multiobjective algorithm for optimizing both ACC and APL on vehicular ad hoc networks to add shortcuts between unconnected networks [34]; this algorithm can be only applied into unconnected wireless networks, but is not suitable for any other kinds of networks.

Given the state-of-the-art methods that optimizing ACC, APL or ACC/APL [3, 4, 23–31], most of them are based on the heuristic tactics, which may inevitably fall into local minimum. Even for the single objective optimization where the complexity is smaller, the effectiveness cannot be guaranteed, especially in large-scale networks. Genetic algorithm and simulated annealing algorithm are difficult to converge [4], and the greedy algorithm can only obtain local optimum [3]. In ref. [34], the multiobjective algorithm for optimizing the small-world property is based on the nondominated sorting genetic algorithm II (NSGA-II), but it is less effective than those single-objective-optimization algorithms, which means the acquired Pareto-optimal front is not the ground truce. In the present study, we retain the advantages of the mentioned algorithms, and form an effective comprehensive algorithm. The new designed algorithm may not only output results with more effectiveness, but also find the Pareto-optimal front more efficiently.

In the present study, we propose a novel algorithm to optimize small-world property based on a multiobjective evolutionary algorithm with decomposition, termed as MOEA/D-SW, which simultaneously optimize the two objectives, ACC and APL. In general, the present study consists of three contributions: (1) We formulize the promotion of small-world property as a mathematical problem, and thus we propose specialized optimization framework that minimizes APL and maximizes ACC separately and simultaneously. The framework is also beneficial for interpreting classic social theories. (2) We propose an algorithm for optimizing small-world property based on MOEA/D-SW, which can be more effective than traditional algorithms despite of single-objective or multi-objective optimization. (3) The optimization results of MOEA/D-SW are further explained to find specific paths for optimizing different objective functions. The present study also explore the optimization mechanisms under different network structures.

In the following, we present a literature review to the generation or transformation of small-world networks, and propose the objective functions to be optimized in Section 2. Section 3 describes the proposed MOEA/D-SW in detail. We conduct experiments with the proposed method on different kinds of networks in Section 4, Conclusion and discussion are presented in Section 5.

## 2. Related background

### 2.1 Optimization of small-world property

“Collective dynamics of ‘small-world’ networks” written by Watts and Strogatz was the first study to define the structure of small-world networks [1]. It describes a dynamic model of edge rewiring process (WS model), where a probability *p* adjusts for the long-distance edges rewired from a regular, locally connected structure, to a completely randomized state. In this progress, there exists an intermediate state with high ACC, like regular networks, and low APL, like random networks. This state is referred as the small-world feature.

One drawback of WS model is that it may lead to isolated nodes in the rewiring process. To solve this problem, Newman and Watts chose to add edges in the dynamics (referred as NW model) [21]. The process of adding edges is based on randomization, which cannot guarantee the effectiveness of the resulting small-world network when the number of edges is limited. This research has prompted a list of studies focusing on adding shortcuts to improve diffusion efficiency [23–28].

Among the methods of optimizing the small-world property by adding edges, evolutionary algorithms have gradually become more popular, which are parallel in nature and are effective in solving combinatorial explosion problems. In the research proposed by Du et al. [4], an index of ACC/APL is proposed to quantifying the small-world feature, and a genetic algorithm (GA) and a greedy algorithm (GR) are proposed to optimize the index. Compared with different heuristic operators, GA outputs the best result, and has been also proved to be effective in promoting ACC [4]. GR is the most time-saving, but can only achieve local optimum. Du et al. proposed a memetic algorithm (MA) that can minimize APL making the network a smaller world [3]. The memetic strategy combines the strength of GA and GR, which has proved to be more effective than the classic shortcut-adding algorithms including Average Shortest Path Distance Minimization [35], EdgeEffect Algorithm [36], Fast MinAPL algorithm [37], Path Screening Algorithm [38], although the time complexity of MA is higher. These studies have laid the foundation for optimizing the generating process of small-world networks. The present study may also apply some of these evolutionary techniques in the proposed algorithm, e.g. Genetic Operator, Tournament Selection and Local Search.

### 2.2 Objective functions

A small-world network can be characterized as low APL and high ACC [1]. APL measures the average shortest distance between all pairs of nodes, and is formulated as $\text{APL}=(1/N(N-1))\sum_{i,j}d(i,j)$, where *N* is the network size and *d*(*i*, *j*) is the geodesic distance between nodes *i* and *j*. ACC is the average value of the local clustering coefficient CC_*v*_ ($\text{ACC}=\frac{\sum_{v}{\text{CC}}_{v}}{n}$), where *CC*_*v*_ = |*E*(Γ_*v*_)|/(*k*_*v*_ (*k*_*v*_ − 1)). |*E*(Γ_*v*_)| is the number of edges among node *v*’s neighbors, (*k*_*v*_ (*k*_*v*_ − 1)) is the number of pairs of nodes among node *v*’s neighbors. In the present study, we aim to minimize APL and maximize ACC simultaneously by adding edges. Specifically, given a connected network with the size of *N* and the number of edges ‖*E*‖, we aim to add *k* edges to reduce APL and increase ACC. The corresponding objective functions can be formulated as Eq (1).


$$\begin{array}{l} \left\{\begin{array}{c} \text{min}\;\text{APL}\\ \text{min}-\text{ACC} \end{array}\right.\\ s.t.\;k\;\text{is}\;\text{fixed} \end{array}$$ (1)

### 2.3 Multiobjective optimization

The present study aims to simultaneously decrease APL and increase ACC, which is a multiobjective optimization problem (MOP). MOP can be described as Eq (2), where *x* is the decision vector, *m* is the number of objective functions, and Ω is the decision space.


$$\begin{array}{l} \text{min}\;\;F(x)={\left(f_{1}(x),\ldots ,f_{m}(x)\right)}^{T}\\ s.t.\;x\in \Omega \end{array}$$ (2)

In a minimization problem for each objective, a decision vector *x*_*A*_ dominates another vector *x*_*B*_ (*x*_*A*_ ≻ *x*_*B*_) if and only if they follow the rule of Eq (3).


$$\forall i=1,2,\ldots ,m\;\;f_{i}(x_{A})\leq f_{i}(x_{B})\wedge \exists j=1,2,\ldots ,m\;\;f_{j}(x_{A})\leq f_{j}(x_{B})$$ (3)

Then a decision vector x* becomes the Pareto-optimal solution if there are no any other vectors that dominate x*. The Pareto-optimal set is formulated as Eq (4).


$$PS^{*}≜\left\{x^{*}\in \Omega |\neg \exists x\in \Omega ,\;x≻x^{*}\right\}$$ (4)

The corresponding objective functions of the Pareto-optimal set is the Pareto-optimal front described as Eq (5).


$$PF^{*}=\left\{F(x^{*})={\left(f_{1}(x^{*}),\ldots ,f_{m}(x^{*})\right)}^{T}|x^{*}\in PS^{*}\right\}$$ (5)

Multiobjective optimization algorithms aim to find the set of nondominated solutions that approximates the true Pareto-optimal front. Much attention has been paid on the employment of evolutionary knowledge in the design of multiobjective optimization algorithms, such as Pareto archived evolutionary strategy (PAES) [39], strength Pareto evolutionary algorithm II (SPEA-II) [40], or nondominated sorting genetic algorithm II (NSGA-II) [41]. Among these studies, the Multiobjective Evolutionary Algorithm based on Decomposition (MOEA/D) [42] is the state-of-the-art method that decomposes MOP into several scalar optimization subproblems and simultaneously optimizes them. This algorithm has been shown to be effective in solving complex multiobjective optimization problems, especially in the field of network science [43–47]. Because of its high performance, we proposed a novel algorithm to multiply improve the small-world property based on MOEA/D (referred as MOEA/D-SW).

With regard to the optimization of small-world property, NSGA-II has been applied to adding shortcuts between communities, which output the best results compared with different multiobjective optimization strategies [34]. Benefiting from the strength of genetic technique, NSGA-II displays powerful spatial search capabilities. The present algorithm may also employ the genetic operator. The difference is that MOEA/D-SW includes the strength of local search, which significantly improves the capability of short-distance search. Moreover, the mechanism of decomposition is completely different from that of NSGA-II, which is more appropriate for the optimization of small-world property.

## 3. The algorithm

### 3.1 Framework of MOEA/D-SW

The idea behind MOEA/D is that the approximation of Pareto-optimal front can be decomposed into a list of scalar optimization subproblems [43]. The algorithm generates a population of solutions for each subproblem, and there exist a list of neighborhood relations among these subproblems, which are calculated by the distances between their aggregation coefficient vectors. With such an operation, two neighboring solutions are similar, while those pairs of long-distance solutions are quite different. At each generation, each subproblem is optimized by using information from its neighboring subproblems.

There are several approaches for constructing aggregation functions, such as Tchebycheff Approach or Weighted Sum Approach [48]. Since we cannot predict whether the Pareto-optimal front is concave, Weighted Sum Approach is not appropriate here, and thus we employ Tchebycheff approach in MOEA/D-SW. Based on the Tchebycheff approach, the scalar optimization problem is described as Eq (6),

$$\begin{array}{l} \text{min}\;\;g^{te}(x|\lambda ,z^{*})=\underset{1\leq i\leq m}{\text{max}}\;\lambda_{i}|f_{i}(x)-z_{i}^{*}|\\ s.t.\;x\in \Omega \end{array}$$ (6)

where $z^{*}=(z_{1}^{*},\ldots ,z_{m}^{*})$ submits to $z_{i}^{*}=\text{min}\{f_{i}(x)|x\in \Omega \}$ for each objective function *i*. In this approach, there exists a weight *λ* that can make each Pareto-optimal vector x* to be optimal in Eq (6), and altering the weight vector that adjusts for neighborhood relations can output different Pareto-optimal solutions. Since *g*^*te*^(*x*|*λ*, z*) is continuous of the weight *λ*, when *λ*_*i*_ is close to *λ*_*j*_, the solution of *g*^*te*^(*x*|*λ*_*i*_, z*) then should be similar to *g*^*te*^(*x*|*λ*_*j*_, z*). One of the motivations behind MOEA/D is that the information of solutions of *g*^*te*^ with different weight vectors which are close to that of *λ*_*j*_ can be helpful in promoting *g*^*te*^(*x*|*λ*_*i*_, z*). This provides an opportunity for the local search.

Algorithm 1 presents MOEA/D-SW framework. Some necessary parameters and the adjacent matrix are first input. A population consisting of *S*_pop_ solutions is initialized by Initial_Population(). Under the maximum iteration *G*_max_, a repeated process is conducted to search for the best solution. In this process, we conduct the genetic operation for each solution. For each solution *j*, we detect its neighboring solutions by finding its nearest corresponding weight vectors. Then we operate crossover and mutation in the Genetic_Operation() on the detected neighboring solutions to generate an offspring population. Update_Population() employs the Tchebycheff approach and find optimal solutions among the neighboring solutions and offspring solutions. Next, the operator removes the less optimal solutions. In the next step, we use the function Local_Search() to find the local optimized chromosome. Finally, we output the optimal set of solutions.

**Algorithm 1**. Framework of MOEA/D-SW

1. Input: maximum number of generations *G*_max_; population size *S*_pop_; size of neighboring population *S*_neighbor_; probability of crossover *P*_c_; a pair of weight vectors which follows the uniform spread: *λ*^1^, *λ*^2^; the initial network adjacency matrix *A*;

2. *P* ← Initial_Population(*S*_pop_);

3. For *i* = 1: 1: *G*_max_

4.  For *j* = 1: 1: *S*_pop_

5.   Find *P*_*j*_’s neighboring population *P*_neighbor_ consisting of *S*_neighbor_ vectors based on *λ*^1^ and *λ*^2^

6.   *P*_offspring_ ← Genetic_Operation(*P*_neighbor_, *P*_c_);

7.   *P* ←Update_Neighbor(*P*_neighbor_, *P*_offspring_, *λ*^1^, *λ*^2^);

8.  End For

9.  *P* ← Local_Search(*P*);

10. End For

11. Output: an external population

### 3.2 Representation

**Fig 1:**
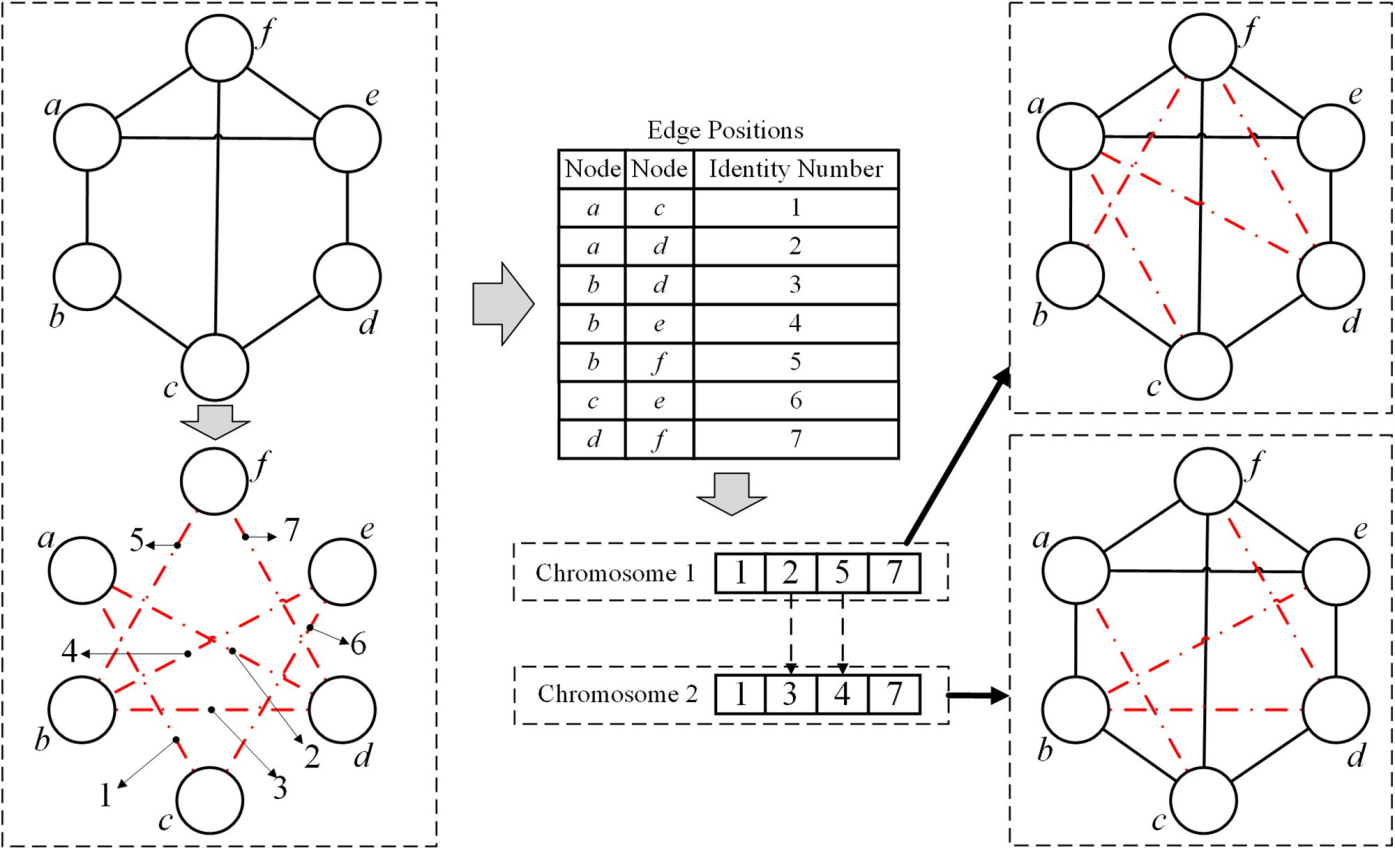
Illustration of the representation. The left part is the topology of the original network and its positions of disconnected edges. The middle part is the identity number corresponding to the disconnected edges, and an illustration of chromosomes. The right part is the topological graph corresponding to the two chromosomes. Solid lines denote the existing edges, and dashed lines denote the edges to be added.

Each solution is represented by a chromosome coded by genes. In the present study, we aim to find the pairs of unconnected nodes to be added in order to optimize the small-world property. Therefore, we first find out the positions of those unconnected pairs of nodes, and assign an identity number for each position. The solution can then be written as a series of the corresponding identity numbers. The length of the chromosome is equaling to the number of added edges, and each gene denotes the corresponding unconnected pair of nodes. An illustration of the representation is shown in Fig 1. If we change the 2^nd^ gene from 2 to 3 and the 3^rd^ gene from 5 to 4, then the added edges between *a* and *d* and between *b* and *f* are respectively changed to the positions between *b* and *d* and between *b* and *e*.

In the initialization, a population consisting of *S*_pop_ chromosomes is generated, and we randomly assign a set of identity numbers to each chromosome. We also compute the Euclidean distances between each pair of vectors and output the *T* closest weight vectors. For each object *i* = 1 and 2 (1 for ACC, 2 for APL), we generate *T* pairs of closest weight vectors *λ*_1_ and *λ*_2_. Increasing the number of weight vectors may output a smoother PF, but sacrifices the time complexity. Consistent to ref. [45], the present study sets *T* = 100, which is capable of finding a satisfactory outcome.

### 3.3 Genetic operation

Genetic operation is the primary function of Genetic Algorithm, where crossover and mutation operations are employed. In this part, crossover procedure is conducted with the probability of *P*_c_ and the mutation procedure runs with 1 − *P*_c_.

**Fig 2:**
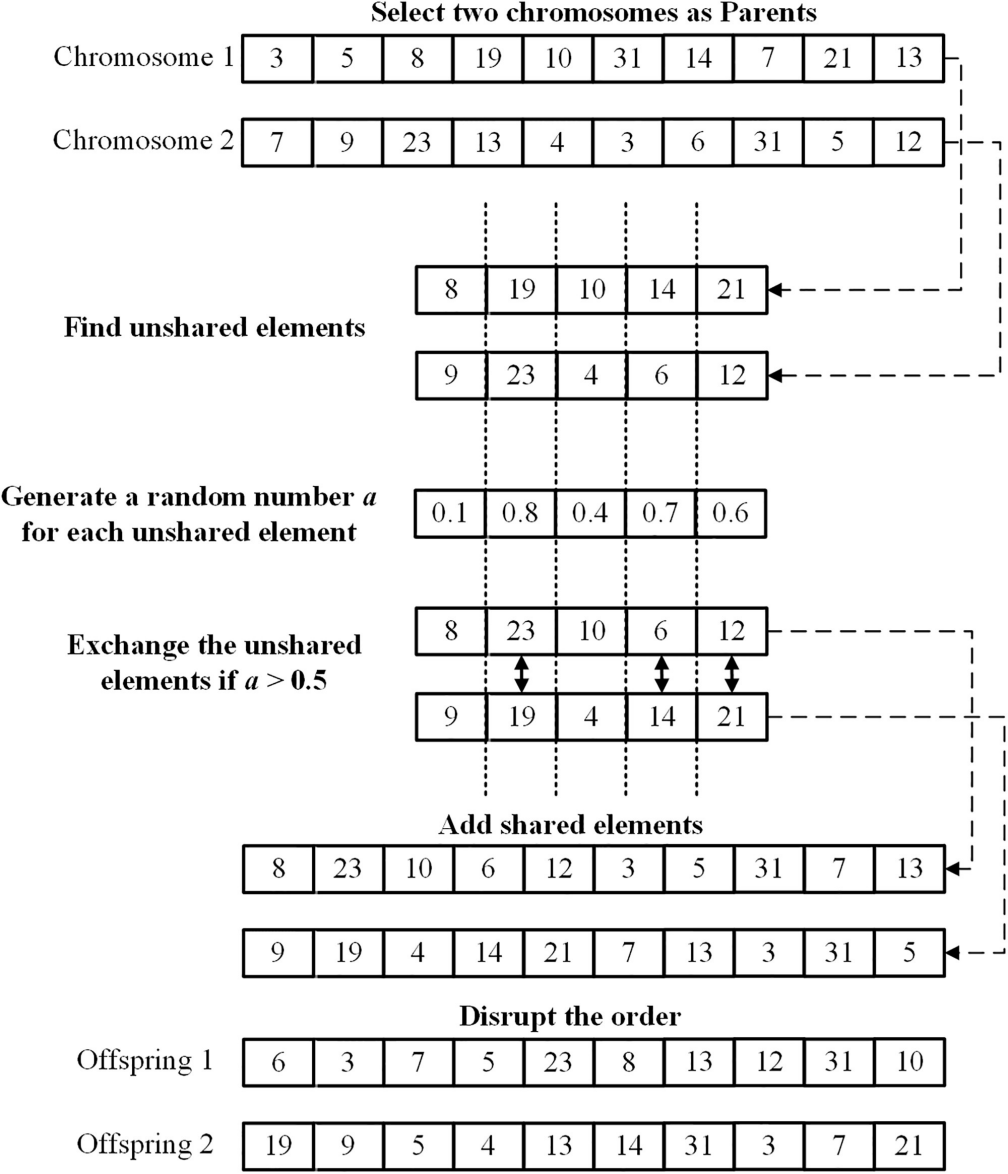
Illustration of crossover. We check every pair of uncommon genes of the pair of parental chromosomes, if the generated random number *a* is smaller than 0.5, the gene remains unchanged; and if *a* is bigger than 0.5, the corresponding genes are exchanged. Finally, we add the shared elements to new offspring chromosomes and disrupt their gene order.

In the crossover procedure, we first randomly select two chromosomes from the focal chromosome’s neighboring population detected based on the nearest corresponding weight vectors as parental chromosomes. The shared elements of offspring chromosomes remain unchanged, and the crossover is conducted only for those uncommon elements which differ between the two parental chromosomes. For each pair of unshared elements, a random number *a* (0 ≤ *a* ≤ 1) is generated. If *a* > 0.5, the corresponding pair of unshared genes are exchanged; otherwise, they remain unchanged. In the next step, we add those shared genes on the two new links. Finally, we disrupt the gene order of the two chromosomes and output two offspring solutions. An illustration of the crossover is shown in Fig 2. The identity numbers 3, 5, 31, 7, 13 are shared elements of both selected chromosomes and they remain unchanged. For the other five pairs of unshared elements, the 2^nd^, 4^th^ and 5^th^ genes are swapped between the two parental chromosomes because *a* is bigger than 0.5 for them.

During mutation, we randomly choose a chromosome from the neighboring population. We randomly generate a number *x* from 0 to the number of added edges. We then randomly select *x* genes to be mutated, i.e. a different identity number is randomly selected to replace that gene.

### 3.4 Local search

In order to improve the algorithm efficiency, we introduce a local search in MOEA/D-SW. The local search operator has been proved to be capable of decreasing useless explorations and accelerating the iteration convergence in the field of network science [49, 50]. Given the updated population derived by previous operations, we check each gene for each solution and find out whether assigning the corresponding edge’s neighbor’s value to it can simultaneously lead to a higher ACC and a lower APL. In this part, an edge’s neighbor is the edge sharing one of the same nodes. If the replacement is able to improve both the two objective functions, then we accept the new assignment. In order to eliminate the endogeneity, we check each gene in a random order.

**Algorithm 2**. Local Search

1. Input: the updated population *P*; size of population *S*_pop_; the number of edges to be added *k*;

2. For *i* = 1: 1: *S*_pop_

3.  Generate a sequence (Seq) with a disordered list from 1 to *k*;

4.  For *j* = 1: 1: *k*

5.   Find out *P*(*i*, Seq(*j*))’s neighbors *y* and the number of these neighbors *s*;

6.   Generate a population *C* consisting of *s* chromosomes of *P*(*i*) and generate a null set *P*^new^;

7.   For *l* = 1: 1: *s*

8.    *C*(*l*, Seq(*j*)) = *y*(*l*);

9.    If ACC(*C*(*l*)) > ACC(*P*(*i*)) & APL(*C*(*l*)) < APL(*P*(*i*))

10.     Add C(*l*) to *P*^new^

11.    end If

12.   End For

13.   Randomly select a chromosome from *P*^new^ to replace *P*(*i*) if *P*^new^ is not a null set;

14.  End For

15. End For

16. Output the new population *P*

### 3.5 Update

After performing the local-search operator, we update the population based on the updating procedure. We first update *z*, i.e. for each object *j* = 1 and 2, we assign *z*_*j*_ = *f*_*j*_(*y*′) if *z*_*j*_ = *f*_*j*_(*y*′). Then we update the neighboring solutions, i.e. for each object *j* = 1 and 2, we let *x*_*j*_ = *y*′ if *g*^*te*^ (*x*_*j*_|*λ*_*j*_, *z*) ≥ *g*^*te*^ (*y*′|*λ*_*j*_, *z*). Further, we remove the vectors dominated by *y*′, and add those vectors that dominate *y*′ as well.

When the procedure reaches its maximum iteration, MOEA/D-SW may output a list of optimal solutions, each of which corresponds to a specific tradeoff between ACC and APL. The solution set may diverse the shortcut adding ways, which provides a hierarchical structure of the optimal network.

## 4. Experiments

In this section, we test the performance of MOEA/D-SW on a regular network and a random network. We also conduct the experiment on various real-world networks and interpret the experimental results in the social context. Table 1 shows the necessary parameters used in the experiments. Specifically, the larger *G*_max_, *S*_pop_, or *S*_neighbor_ help increase precision but sacrifice the time complexity. Therefore, we set them to moderate values that can form satisfactory solutions by MOEA/D, consistent to ref. [45]. *P*_c_ adjusts for the allocation between the long-distance and short-distance search, and the value of 0.1 for *P*_c_ can output the best performance for MOEA/D-SW as shown in the appendix. The procedure is carried out by Matlab on a 32-core workstation with 128 GB memory.

**Table 1:** Parameters in the experiment.

Parameter	Meaning	Value
*G*_max_	Maximum number of generations	100
*S*_pop_	Size of population	100
*S*_neighbor_	Size of neighboring population	15
*P*_c_	Probability of Crossover	0.1

### 4.1 Results for computer-generated regular and random networks

We first respectively carry out the proposed algorithm on a regular network and a random network generated by computers for 10 times. The two networks consist of 30 nodes with the average degree of 8. Among the state-of-the-art algorithms that aim to optimize the small-world property, 4 single-objective optimization model including the memetic algorithm (MA-SW) proposed by Du et al. [3], the genetic algorithm (GA-SW) and the greedy algorithm (GR-SW) proposed by Du et al. [4], and the typical Newman’s and Watts’s proposed model in 1999 (NW model) [21] are popular ones. The present study aims to compare the effectiveness of MOEA/D-SW with the four algorithms. As mentioned in Section 2, MA is effectively in minimizing APL, GA performs the best on promoting ACC/APL and ACC, GR is the most time-saving, while NW is the most classic. We also compare the result of MOEA/D-SW with NSGA-II, because it has been already applied to adding shortcuts, and it performs the best among different multiobjective optimization strategies [34].

**Fig 3:**
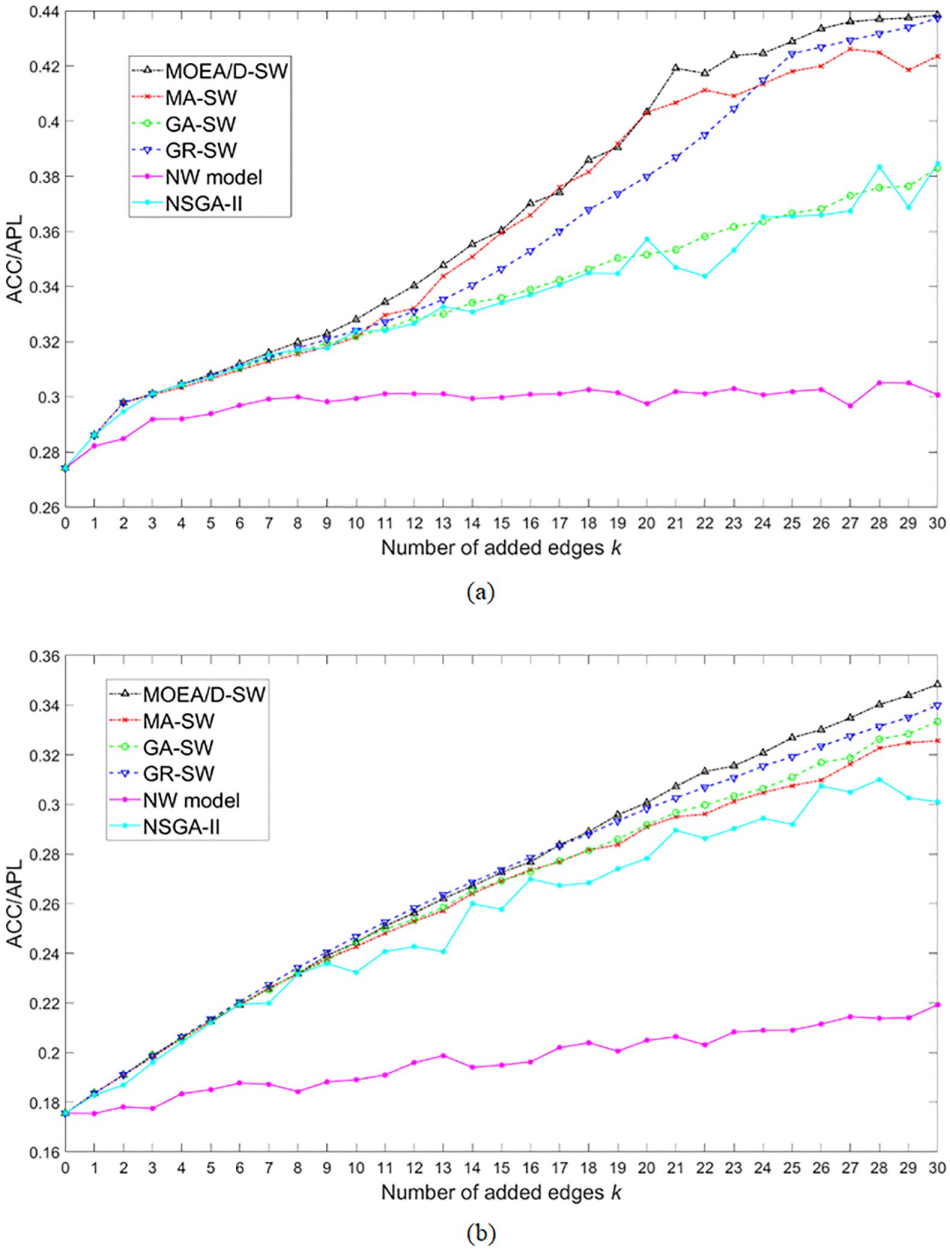
The mean value of the maximum ACC/APL for each number of added edges *k* for 10 runs acquired by MOEA/D-SW, MA-SW, GA-SW, GR-SW, NW model, and NSGA-II. (a) and (b) are the results on the regular and the random networks, respectively.

According to Du et al. [4], the small-world property can be generally formulated as ACC/APL, and we thus compare the mean value of the maximum ACC/APL derived by MOEA/D-SW with the results derived by other methods. Fig 3 shows the optimal ACC/APL for each number of added edges *k* acquired by the 6 algorithms on the regular network and the random network. Generally, ACC/APL has the same tendency among the regular network and the random network, i.e. it increases as *k* increases. Among the 6 algorithms, MOEA/D-SW always performs the best, from which can be concluded that MOEA/D-SW can effectively solve the problem of optimizing the small-world property. GR-SW performs relatively steady on both the two network structures, while MA-SW is more limited on the random network because of the local optimism. GA-SW performs fare well on the random network but is limited on the optimization process on the regular network. NSGA-II performs less effectively on the single-objective optimization. Since NW model is based on the principle of randomly adding edges, it cannot guarantee the effectiveness of improving the small-world property. Tables 2 and 3 show statistical tests of ACC/APL among different algorithms, and we can find MOEA/D-SW is significantly effective than its peers.

**Table 2:** Statistical tests of ACC/APL on the regular network.

Algorithm	MOEA/D-SW	MA-SW	GA-SW	GR-SW	NW model	NSGA-II
MOEA/D-SW	-	5.783***	6.381***	5.760***	7.843***	6.317***
MA-SW	-	-	5.949***	1.730	7.793***	5.899***
GA-SW	-	-	-	-5.160***	9.321***	1.982
GR-SW	-	-	-	-	7.333***	5.245***
NW model	-	-	-	-	-	-9.171***
NSGA-II	-	-	-	-	-	-

Note:

***means the corresponding p<0.001;

**means the corresponding p < 0.01; and

*means the corresponding p < 0.05

**Table 3:** Statistical tests of ACC/APL on the random network.

Algorithm	MOEA/D-SW	MA-SW	GA-SW	GR-SW	NW model	NSGA-II
MOEA/D-SW	-	5.594***	5.590***	2.595*	10.911***	6.905***
MA-SW	-	-	-4.282***	-7.868***	12.018***	7.173***
GA-SW	-	-	-	-8.558***	11.787***	7.141***
GR-SW	-	-	-	-	11.600***	8.084***
NW model	-	-	-	-	-	-12.112***
NSGA-II	-	-	-	-	-	-

Note:

***means the corresponding p<0.001;

**means the corresponding p < 0.01; and

*means the corresponding p < 0.05

**Fig 4:**
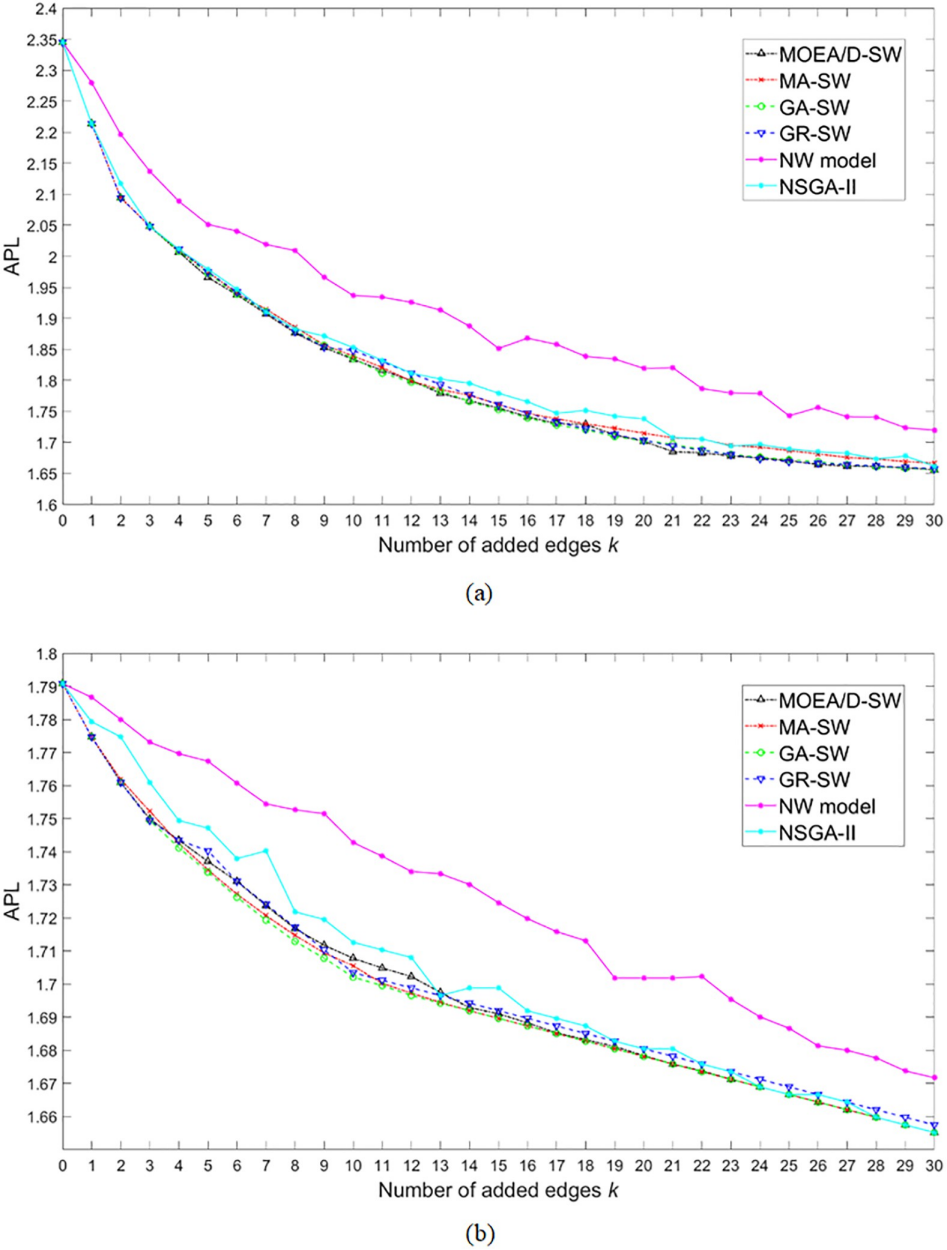
The mean value of the minimum APL for each number of added edges *k* for 10 runs acquired by MOEA/D-SW, MA-SW, GA-SW, GR-SW, NW model, and NSGA-II. (a) and (b) are the results on the regular and the random networks, respectively.

We also present the results of the minimum APL among the 6 algorithms in Fig 4, and we can find MOEA/D-SW and GA-SW perform the best. GR-SW and MA-SW are less effective, but they are nearly coincident with MOEA/D-SW and GA-SW. NSGA-II performs better than NW model, but it is still limited to the single-objective optimization. The specific statistical tests can be seen from Tables 4 and 5.

**Table 4:** Statistical tests of APL on the regular network.

Algorithm	MOEA/D-SW	MA-SW	GA-SW	GR-SW	NW model	NSGA-II
MOEA/D-SW	-	-7.384***	-1.384	-4.148***	-26.058***	-10.523***
MA-SW	-	-	7.995***	3.110**	-22.730***	-5.167***
GA-SW	-	-	-	-2.850**	-25.598***	-8.910***
GR-SW	-	-	-	-	-26.983***	-7.289***
NW model	-	-	-	-	-	23.657***
NSGA-II	-	-	-	-	-	-

Note:

***means the corresponding p<0.001;

**means the corresponding p < 0.01; and

*means the corresponding p < 0.05

**Table 5:** Statistical tests of APL on the random network.

Algorithm	MOEA/D-SW	MA-SW	GA-SW	GR-SW	NW model	NSGA-II
MOEA/D-SW	-	3.474**	4.301***	-2.239*	-19.523***	-6.128***
MA-SW	-	-	3.370**	-6.238***	-17.576***	-6.563***
GA-SW	-	-	-	-9.894***	-17.189***	-6.482***
GR-SW	-	-	-	-	-16.504***	-4.154***
NW model	-	-	-	-	-	16.401***
NSGA-II	-	-	-	-	-	-

Note:

***means the corresponding p<0.001;

**means the corresponding p < 0.01; and

*means the corresponding p < 0.05

**Fig 5:**
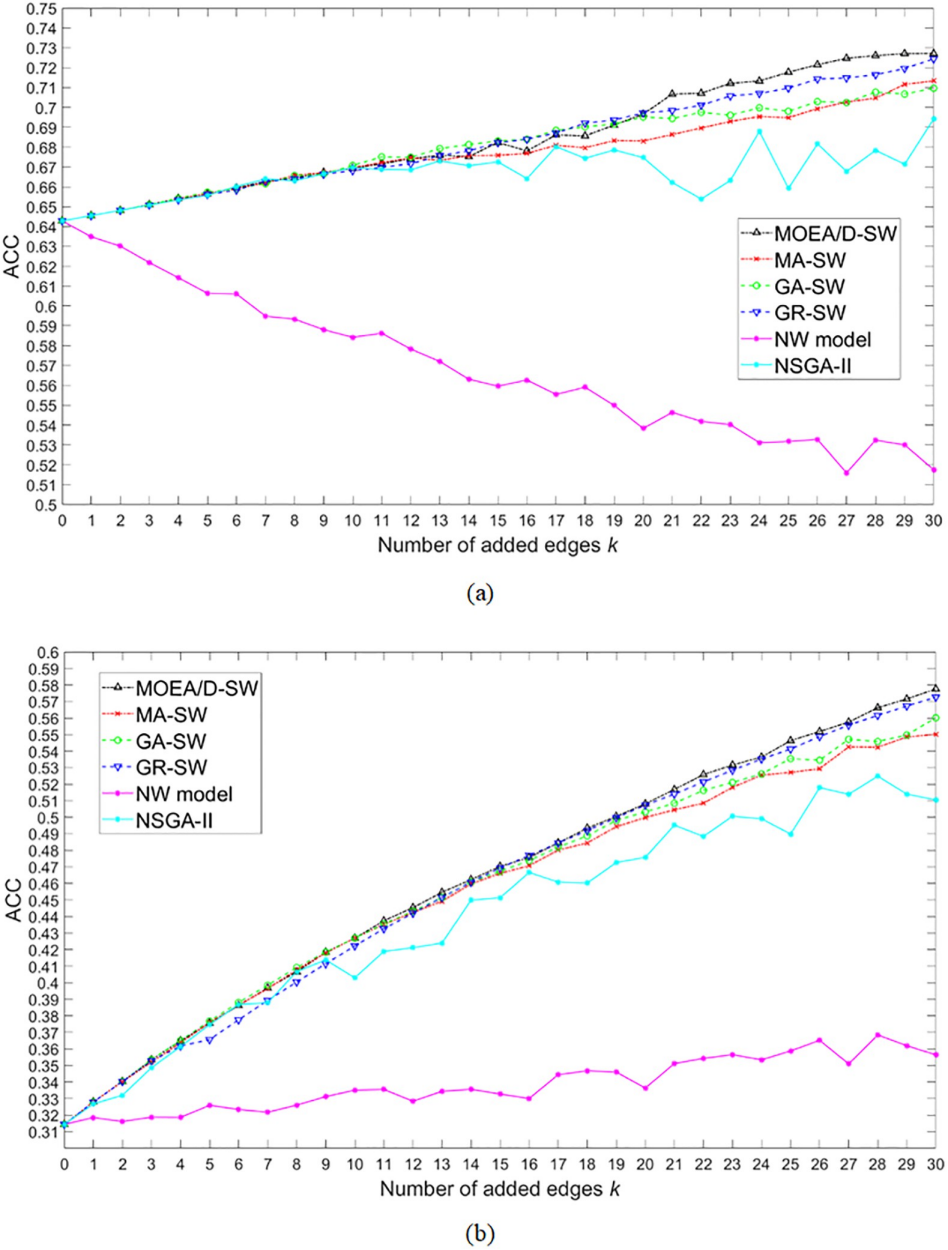
The mean value of the maximum ACC for each number of added edges *k* for 10 runs acquired by MOEA/D-SW, MA-SW, GA-SW, GR-SW, NW model, and NSGA-II. (a) and (b) are the results on the regular and the random networks, respectively.

Fig 5 shows the results of the optimal ACC among the 6 algorithms. MOEA/D-SW performs the best on promoting ACC, GR-SW performs the second, GA-SW performs the third, MA-SW performs the forth, NSGA-II performs the fifth, while NW model cannot effectively optimize ACC. Same conclusions can be also found from the statistical tests in Tables 6 and 7.

**Table 6:** Statistical tests of ACC on the regular network.

Algorithm	MOEA/D-SW	MA-SW	GA-SW	GR-SW	NW model	NSGA-II
MOEA/D-SW	-	4.766***	2.810**	2.673*	10.802***	4.725***
MA-SW	-	-	-4.085***	-5.004***	11.577***	4.400***
GA-SW	-	-	-	-2.521*	11.655***	5.469***
GR-SW	-	-	-	-	11.051***	4.917***
NW model	-	-	-	-	-	-12.714***
NSGA-II	-	-	-	-	-	-

Note:

***means the corresponding p<0.001;

**means the corresponding p < 0.01; and

*means the corresponding p < 0.05

**Table 7:** Statistical tests of ACC on the random network.

Algorithm	MOEA/D-SW	MA-SW	GA-SW	GR-SW	NW model	NSGA-II
MOEA/D-SW	-	5.037***	4.166***	6.622***	11.613***	7.101***
MA-SW	-	-	-5.790***	-2.778**	12.372***	7.615***
GA-SW	-	-	-	-1.415	12.276***	7.839***
GR-SW	-	-	-	-	11.237***	5.977***
NW model	-	-	-	-	-	-12.627***
NSGA-II	-	-	-	-	-	-

Note:

***means the corresponding p<0.001;

**means the corresponding p < 0.01; and

*means the corresponding p < 0.05

**Fig 6:**
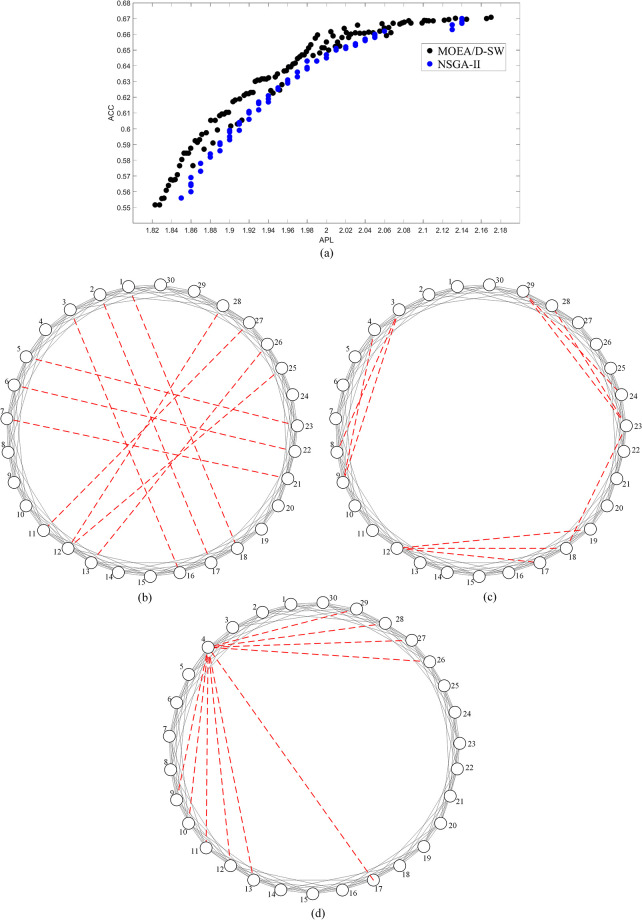
The Pareto-optimal fronts on the regular network with 10 added edges and its corresponding solutions. (a) is the results of Pareto-optimal front, where the black line represents the results acquired by MOEA/D-SW, and the blue line represents the results acquired by NSGA-II, (b) is the solution with the minimum APL, (c) is the solution with the maximum ACC, (d) is the solution with the maximum ACC/APL. The solid lines represent edges of the initial network, the dashed lines represent the added edges.

Fig 6(a) presents the Pareto-optimal fronts derived by MOEA/D-SW and NSGA-II on the regular network with the number of added edges *k* = 10, from which we can find that the two Pareto-optimal fronts have the hierarchical structure. However, MOEA/D-SW is more optimal than NSGA-II as shown in Fig 4(a). Each point denotes a unique solution of adding shortcuts. Corresponding to the solutions of MOEA/D-SW in Fig 4(a), we present three topologies as described in Fig 6(b)–6(d) that respectively output the minimum APL (APL = 1.823, ACC = 0.551), the maximum ACC (APL = 2.170, ACC = 0.671) and the maximum ACC/APL (APL = 1.991, ACC = 0.660). These results reflect different strategies corresponding to different objectives. As shown in Fig 6(b), we can find the optimal strategy is to connect the distant nodes if the main objective is to minimize APL. This strategy can significantly decrease the distance between each pair of nodes. If the objective is to maximize ACC, the optimal strategy is to add edges in the neighborhood that helps construct more triangles as shown in Fig 6(c), although it may sacrifice the decline of APL. As shown in Fig 6(d), the optimal strategy takes both distance and triangles into account if we aim to maximize ACC/APL. This strategy is to connect to a hub node from both distant nodes and neighboring nodes. According to Du et al. [51], establishing connections between the hub nodes and the rest of nodes is an efficient way to decrease diameter. Therefore, connecting distant nodes to the hub can effectively decrease APL. When the new added edges link to those neighboring nodes, more triangles may then be constructed, and thus ACC increases as a result. These results suggest that MOEA/D-SW can output a series of effective solutions for different goals.

**Fig 7:**
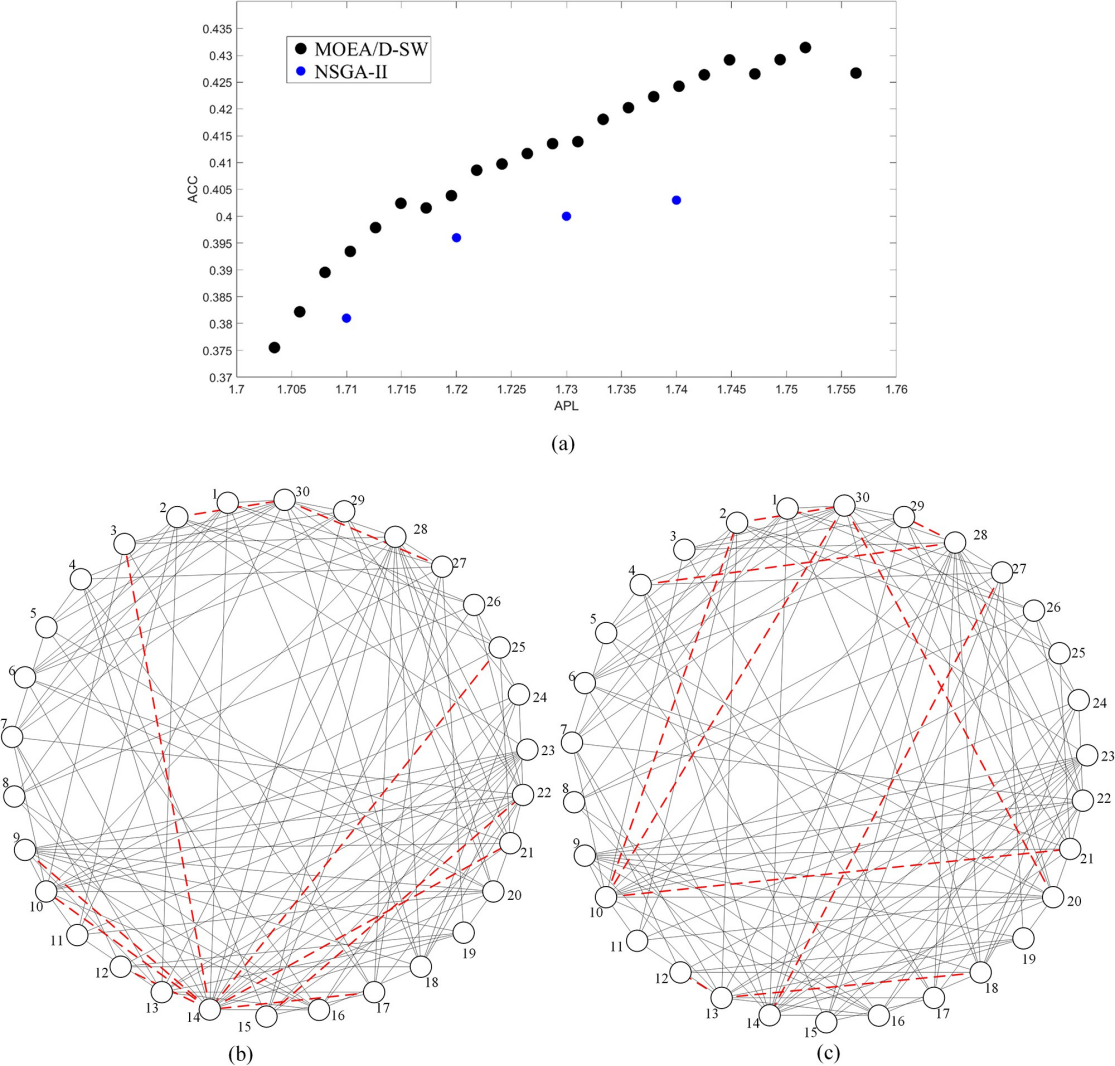
The Pareto-optimal fronts on the random network with 10 added edges and its corresponding solutions. (a) is the results of Pareto-optimal front, where the black line represents the results acquired by MOEA/D-SW, and the blue line represents the results acquired by NSGA-II, (b) is the solution with the minimum APL, (c) is the solution with both the maximum ACC and the maximum ACC/APL. The solid lines represent edges of the initial network, the dashed lines represent the added edges.

Fig 7 presents the Pareto-optimal fronts derived by MOEA/D-SW and NSGA-II on the random network with *k* = 10 and its corresponding solutions. The result of MOEA/D-SW is more effective, and it has a hierarchical structure, while NSGA-II has not. Fig 7(b) and 7(c) correspond to the optimal results of minimum APL (APL = 1.703, ACC = 0.376) and the maximum ACC (APL = 1.752, ACC = 0.431, it is also the result of maximum ACC/APL). When the main object is to minimize APL, most added edges are connected to hub nodes (e.g. nodes 14 and 30), which can decrease the network diameter as shown in Fig 7(b). If we aim to maximize ACC, the optimal strategy is to create as many triangles as possible, shown as Fig 7(c). This solution also outputs the maximum of ACC/APL, because it does not sacrifice much of the decline of APL. Results in Figs 6 and 7 have proved that MOEA/D-SW can find an effective Pareto-optimal front on different network structures.

Furthermore, we compute the assortative index of those solutions derived by MOEA/D-SW to explore the mixing effect of optimizing small-world property with different goals. The assortative index *r* is formulated as Eq (7), where *M* is the edge number, *j*_*i*_, *k*_*i*_ are the corresponding two nodes’ degree at the ends of the *i*^th^ edge [52].


$$r=\frac{M^{-1}\sum_{i}j_{i}k_{i}-{\left[M^{-1}\sum_{i}\frac{1}{2}(j_{i}+k_{i})\right]}^{2}}{M^{-1}\sum_{i}\frac{1}{2}({j_{i}}^{2}+{k_{i}}^{2})-{\left[M^{-1}\sum_{i}\frac{1}{2}(j_{i}+k_{i})\right]}^{2}}$$ (7)

**Fig 8:**
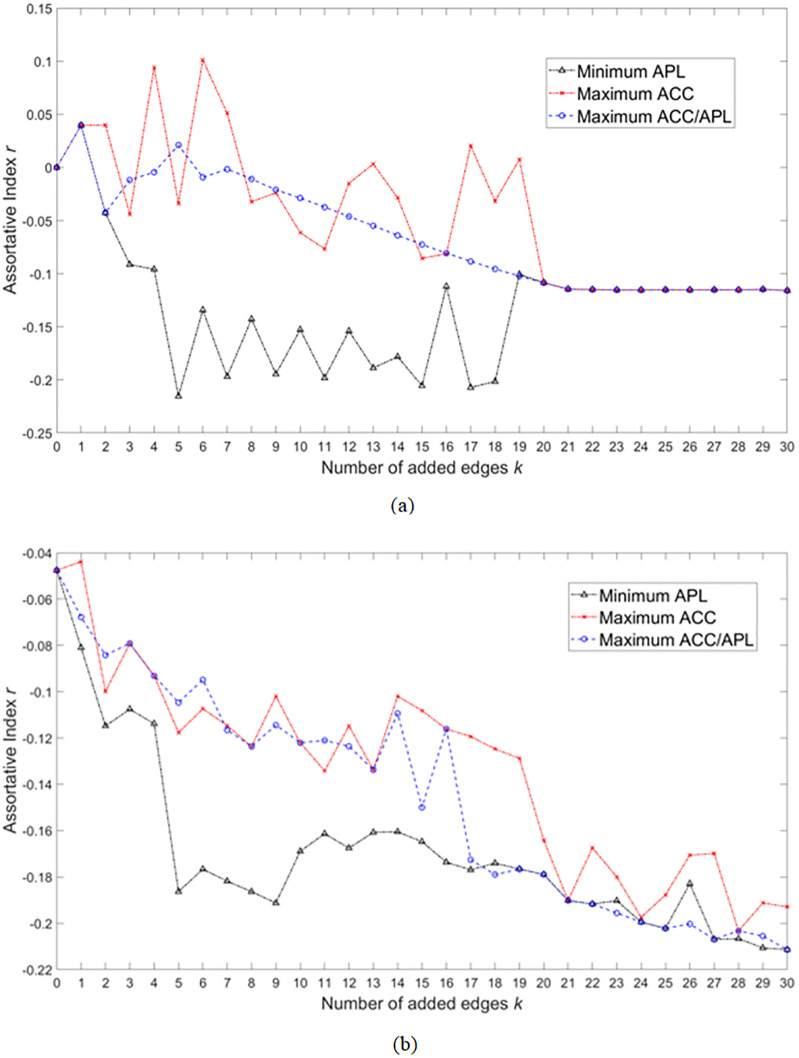
Assortative index *r* for each number of added edges *k* with the solutions of minimum APL, the maximum ACC, and the maximum ACC/APL acquired by MOEA/D-SW. (a) and (b) are the results on the regular and the random networks, respectively.

Fig 8 shows the result of assortative index for the optimized regular network and a random network derived by MOEA/D-SW. All the optimal strategies lead to disassortative structure on both regular and random networks, which suggests that large-degree nodes have a tendency of connecting with small-degree nodes. Among the three strategies, the strategy with the minimum APL is the most disassortative, while the one with the maximum ACC is the least disassortative. This phenomenon suggests that linking nodes with the hub nodes is the common rule for decreasing APL, but may be not for increasing ACC.

### 4.2 Results for real-world networks

In this section, we carry out MOEA/D-SW on five real-world networks and interpret the optimal strategies in the social context.

The five networks are Zachary’s Karate Club network (34 members and 78 social connections) [53], Bottlenose Dolphins network (62 dolphins and 159 ties) [54], American College Football network (115 teams and 616 edges) [55] and two networks of the company colleagues C_1_ (70 nodes and 272 social ties) and C_2_ (193 nodes and 887 social ties) [56].

Table 8 shows the mean results of APL, ACC and ACC/APL of the optimal networks with different goals and number of added edges over 10 runs. We find that MOEA/D-SW is capable of finding different substantial solutions according to the different preference of the optimization. Given the three classic goals of measuring the small-world property, the minimum APL, the maximum ACC, and the maximum ACC/APL, most of the output solutions under these three goals are unique. Several solutions under the goals of maximum ACC and the maximum ACC/APL are similar, which suggests that the index of ACC/APL proposed by Du et al. [4] is more inclined to ACC.

**Table 8:** APL, ACC and ACC /APL of the optimal networks with different goals and number of added edges.

Network	Index	Number of added edges *k* = 5			Number of added edges *k* = 10			Number of added edges *k* = 30		
		Minimum APL	Maximum ACC	Maximum ACC/APL	Minimum APL	Maximum ACC	Maximum ACC/APL	Minimum APL	Maximum ACC	Maximum ACC/APL
Karate	APL	**2.102**	2.198	2.148	**1.970**	2.075	2.075	**1.808**	1.850	1.808
	ACC	0.612	**0.715**	0.702	0.691	**0.773**	0.773	0.852	**0.852**	0.852
	ACC/APL	0.291	0.325	**0.327**	0.351	0.373	**0.373**	0.471	0.461	**0.471**
Dolphins	APL	**2.787**	3.319	3.021	**2.640**	2.960	2.815	**2.263**	2.377	2.350
	ACC	0.290	**0.414**	0.390	0.288	**0.437**	0.422	0.488	**0.545**	0.539
	ACC/APL	0.104	0.125	**0.129**	0.109	0.148	**0.150**	0.216	0.229	**0.229**
Football	APL	**2.460**	2.503	2.503	**2.432**	2.495	2.495	**2.359**	2.427	2.427
	ACC	0.399	**0.414**	0.414	0.394	**0.421**	0.421	0.378	**0.435**	0.435
	ACC/APL	0.162	0.165	**0.165**	0.162	0.169	**0.169**	0.160	0.179	**0.179**
C_1_	APL	**2.322**	2.390	2.364	**2.235**	2.293	2.278	**1.953**	2.042	1.972
	ACC	0.523	**0.566**	0.566	0.538	**0.598**	0.595	0.628	**0.659**	0.642
	ACC/APL	0.225	0.237	**0.239**	0.241	0.261	**0.261**	0.322	0.323	**0.326**
C_2_	APL	**2.793**	2.871	2.871	**2.737**	2.838	2.790	**2.588**	2.639	2.604
	ACC	0.432	**0.457**	0.457	0.435	**0.471**	0.468	0.479	**0.506**	0.504
	ACC/APL	0.155	0.159	**0.159**	0.159	0.166	**0.168**	0.185	0.192	**0.194**

**Fig 9:**
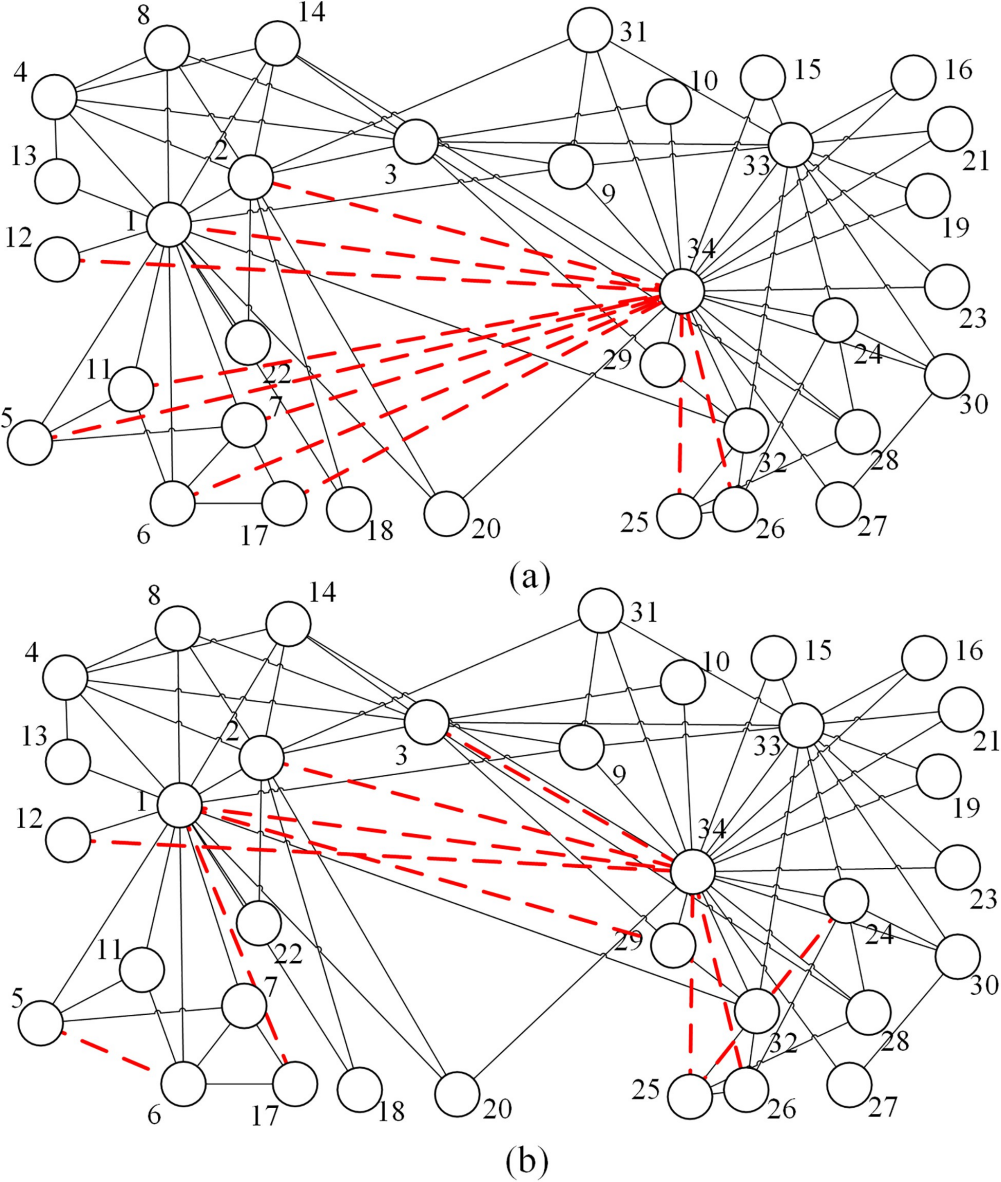
The topologies of edging solution with *k* = 10 on Zachary’s Karate Club network. (a) is the result with the minimum APL, (b) is the result with both the maximum ACC and the maximum ACC/APL. The solid lines represent edges of the initial network, the dashed lines represent the added edges. The network has a strong characteristic of community structure, and it can be divided into two communities (the left part and the right part).

Fig 9(a) and 9(b) respectively present the topologies of edging solution of the minimum APL and maximum ACC or ACC/APL with *k* = 10 on Zachary’s Karate Club network. When the main goal is to decrease APL, connecting other nodes to the-highest-degree node is the perfect way to improve the small-world property as shown in Fig 7(a). Under this strategy, nearly all the nodes are linked to node 34 (except for nodes 4, 8, 13, 18), which means the network diameter is getting more closed to 2 which is a critical diameter [51], i.e. each node can be contacted within only 2 steps. When we give priority to the optimism of ACC, most of the work is to construct more triangles, e.g. connecting nodes 5 and 6 can build triangles 5-6-7, 5-6-11, 5-6-1. Compared with (a) and (b), some added edges (edges between nodes 1 and 34, 2 and 34, 12 and 34, 25 and 34, 26 and 34) exist in both solutions. These edges are critical in both promoting ACC and declining APL.

According to Du et al. [4], if the network has a strong characteristic of community structure, connecting nodes within the same community is an effective way to promote the small-world property. However, the edging method is not appropriate in Zachary’s Karate Club network which is also a typical network with the feature of community structure [55]. This can be attributed to the number of added edges and the density. Actually, adding edges within the same community is more likely to create more triangles, and thus helps increase ACC. However, if the local density of the community is large enough, adding the interior edges may not effectively create more triangles, and then the strategy may become useless. Compared with the optimized network in [4], the interior community is already saturated in Zachary’s Karate Club network that there is no need to add more edges within the community. In summary, the impact of community structure on optimizing small-world property mainly lies in the way of creating more triangles. If the internal community is saturated, the optimization process is less impacted by community structure.

In order to explore the effect of community structure, we ran MOEA/D-SW on the benchmark networks proposed by Lancichinetti et al. [57]. The network consists of 128 nodes with the average degree of 16. All the nodes are evenly assigned to a specific clustering attribute 1, 2, 3 or 4. A mixing parameter *δ* is then introduced to denote the fraction of edges that link nodes between different clusters. A higher *δ* represents a more apparent feature of community structure. Table 9 shows the mean results of APL, ACC and ACC/APL of the optimal networks with different *δ* over 10 runs. We find that there exists a sudden reduction of ACC/APL between *δ* = 0 and *δ* = 1/8 when *k* is not large enough. This is because APL significantly increases as *δ* increases from 0 to 1/8. As *δ* increases to a higher level, the optimal APL gradually decreases, and the gradients of the three indices decreases as well. On the other hand, as the number of added edges increases, the difference between different *δ* gets smaller, which suggests community structure has less impact on the optimization of small-world property with increase of the network density.

**Table 9:** APL, ACC and ACC /APL of the optimal benchmark networks with different mixing parameter.

Number of added edges	Index	*δ* = 0	*δ* = 1/8	*δ* = 2/8	*δ* = 3/8	*δ* = 4/8	*δ* = 5/8	*δ* = 6/8	*δ* = 7/8	*δ* = 1
*k* = 5	APL	1.481	2.443	2.233	2.168	2.136	2.051	2.023	1.974	1.976
	ACC	0.521	0.451	0.372	0.295	0.261	0.202	0.196	0.160	0.146
	ACC/APL	0.352	0.181	0.165	0.135	0.122	0.098	0.096	0.081	0.074
*k* = 10	APL	1.807	2.409	2.216	2.154	2.124	2.041	2.015	1.968	1.971
	ACC	0.522	0.454	0.377	0.300	0.265	0.205	0.199	0.163	0.149
	ACC/APL	0.286	0.184	0.168	0.138	0.124	0.100	0.098	0.083	0.076
*k* = 30	APL	2.478	2.322	2.157	2.106	2.083	2.008	1.985	1.947	1.950
	ACC	0.521	0.464	0.388	0.312	0.279	0.218	0.210	0.175	0.161
	ACC/APL	0.203	0.193	0.175	0.145	0.132	0.107	0.105	0.089	0.082

Table 10 presents the mean percentage of edges added within the same community for different values of *δ* and different goals. When *δ* is small, more edges should be added between different communities in order to minimize APL; but if the goal is to maximize ACC, adding edges within the same community is the best choice; if we aim to maximize ACC/APL, the optimal solution depends on the balance between ACC and APL, and the percentage of edges added within the same community experiences an inverted U change as *δ* increases. When *δ* is large enough, the difference between adding edges within the same community and between different communities decreases, and the result does not show a clear pattern. On the other hand, if *k* is small, adding edges within the same community is an effective way to enhance the small-world property; but if *k* is large, there is no need to add more edges within the community because the internal community is saturated.

**Table 10:** Percentage of edges added within the same community in the optimal benchmark networks with different mixing parameter and goals.

Number of added edges	Goals	*δ* = 0	*δ* = 1/8	*δ* = 2/8	*δ* = 3/8	*δ* = 4/8	*δ* = 5/8	*δ* = 6/8	*δ* = 7/8	*δ* = 1
*k* = 5	Min APL	0	0	0	0	0	0	0	0.04	0.1
	Max ACC	1	1	1	1	1	0.9	0.94	0.66	0.74
	Max ACC/APL	1	1	1	1	1	0.9	0.92	0.64	0.8
*k* = 10	Min APL	0.23	0	0	0	0.01	0.04	0.04	0.06	0.36
	Max ACC	0.78	0.86	0.97	0.89	0.86	0.8	0.69	0.54	0.7
	Max ACC/APL	0.27	0.64	0.97	0.9	0.87	0.8	0.69	0.54	0.71
*k* = 30	Min APL	0.027	0.007	0.020	0.047	0.073	0.123	0.107	0.157	0.253
	Max ACC	0.467	0.687	0.713	0.663	0.753	0.617	0.520	0.460	0.507
	Max ACC/APL	0.297	0.683	0.717	0.667	0.760	0.617	0.520	0.463	0.507

## 5. Conclusion

In the present study, we present a multi-objective optimization model to maximize ACC and minimize APL simultaneously. A novel multiobjective evolutionary algorithm is designed, and has been proved to solve the optimization problem efficiently. The experiment shows that the present method can generate a uniform distribution of solutions with distinct hierarchical levels. Simulations on real-world networks also proves the effectiveness of the presented algorithm, and we find the optimization on networks with the feature of community structure is more remarkable, but community structure has less impact on the optimization when the internal community is triangles-saturated. Adding edges within the same community is helpful for promoting ACC, while adding edges between different communities is beneficial for reducing APL. When the feature of community structure weakens, the difference between adding edges between communities and adding edges within communities decreases. Moreover, an increase of network density may also weaken the impact of community structure on small-world optimization.

In many natural settings, researchers have different requirements on the small-world property, e.g. the set of power grid aims to promote ACC to guard against the risk of power failure, while the main object of transport network is to reduce APL to improve transmission efficiency. The present method can help researchers quickly find the edging solutions according to different goals.

Future research may apply the proposed algorithm to networks that contain both node and edge attributes. On the one hand, node attributes may impact on the formation of networks, and thus we can propose a framework of small-world optimization under the tag-mediated system. On the other hand, the present work is only conducted on nonnegative networks, while the edge attribute, especially the negative edge, has been ignored. Therefore, the optimizations of APL and ACC need to be conducted in signed networks. Further, the optimization on dynamic adaptive networks needs to be focused, where nodes and edges are dynamically updated.

## Supporting information

S1 Fig The mean value of the maximum ACC/APL for each number of added edges *k* and different crossover probability *P*_*c*_ for 10 runs acquired by MOEA/D-SW on the regular network. The different curves represent results derived from different *P*_*c*_. We can find the algorithm performs best when *P*_*c*_ = 0.1.
(TIF)

S1 Dataset (RAR)
