# Supplementary figures and images for: A multiobjective evolutionary algorithm for optimizing the small-world property
Source: PLoS One. 2024 Dec 3;19(12):e0313757. doi: 10.1371/journal.pone.0313757 (PMC11614276; doi:10.1371/journal.pone.0313757)

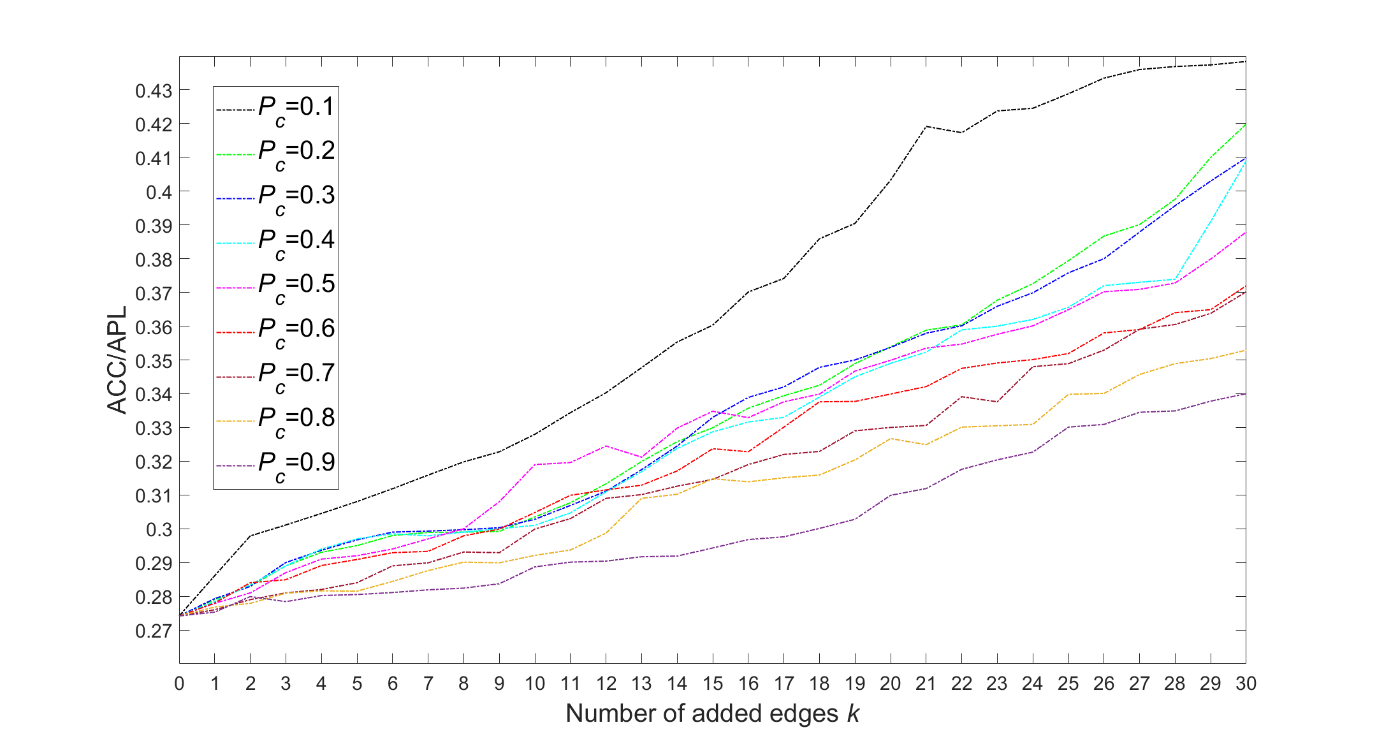

Supplement: S1 Fig — The different curves represent results derived from different Pc. We can find the algorithm performs best when Pc = 0.1. (TIF) [file pone.0313757.s001.tif]
